# Replacement of animal testing by integrated approaches to testing and assessment (IATA): a call for in vivitrosi

**DOI:** 10.1007/s00204-022-03299-x

**Published:** 2022-05-03

**Authors:** Francesca Caloni, Isabella De Angelis, Thomas Hartung

**Affiliations:** 1 Department of Environmental Science and Policy (ESP), Università degli Studi di Milano, Via Celoria 10, 20133 Milan, Italy; 2 Environment and Health Department, Istituto Superiore di Sanità, Viale Regina Elena, 299, 00161 Rome, Italy; 3 Center for Alternatives to Animal Testing (CAAT), Johns Hopkins Bloomberg School of Public Health, Baltimore, MD 21205 USA; 4 CAAT Europe, University of Konstanz, 78464 Konstanz, Germany

**Keywords:** 3Rs, Complexity, Integrated approach, Non-animal methods, Replacement, In toxicology

## Abstract

Alternative methods to animal use in toxicology are evolving with new advanced tools and multilevel approaches, to answer from one side to 3Rs requirements, and on the other side offering relevant and valid tests for drugs and chemicals, considering also their combination in test strategies, for a proper risk assessment.

While stand-alone methods, have demonstrated to be applicable for some specific toxicological predictions with some limitations, the new strategy for the application of New Approach Methods (NAM), to solve complex toxicological endpoints is addressed by Integrated Approaches for Testing and Assessment (IATA), aka Integrated Testing Strategies (ITS) or Defined Approaches for Testing and Assessment (DA). The central challenge of evidence integration is shared with the needs of risk assessment and systematic reviews of an evidence-based Toxicology. Increasingly, machine learning (aka Artificial Intelligence, AI) lends itself to integrate diverse evidence streams.

In this article, we give an overview of the state of the art of alternative methods and IATA in toxicology for regulatory use for various hazards, outlining future orientation and perspectives. We call on leveraging the synergies of integrated approaches and evidence integration from in vivo, in vitro and in silico as true in vivitrosi.

## Introduction

Toxicology has traditionally relied on animal testing as the primary evidence stream. However, the life sciences have advanced offering a continuously expanding portfolio of technologies, mechanistic understanding and data analysis approaches. To answer to the requirements of testing chemicals and products, as well as to follow the 3Rs concept of Replace, Reduce and Refine to incorporate NAM to complement and substitute complex in vivo studies, toxicology is looking to new approaches and strategies (Caloni et al. 2021). This requires considering new promising tools, like spheroids, organoids, and organs-on-chip (Lee and Lee 2020), jointly referred to as Microphysiological Systems (MPS) (Marx et al. 2016, 2020; Roth and MPS-WS Berlin 2019 2021), building relevant in vitro systems, or to combine different tests *in chemico*, in vitro, in silico, to mimic the different cellular and molecular events (Rovida et al. 2015), considering also the increasing attention in risk assessment on the combination of chemical substances and mixtures (Kar and Leszczynski 2019; Hayes et al. 2020).

The concept of Integrated Testing Strategies originated early on out of an ECVAM taskforce with idea of combining methods to replace animal testing (Blaauboer et al. 1999; DeJongh et al. 1999; Blaauboer and Barratt 1999). The emergence of the European REACH legislation and its enormous challenges independently furthered this discussion (Ahlers et al. 2008). In a report (Jaworska and Hoffmann 2010) commissioned by the Center for Alternatives to Animal Testing (CAAT), opportunities to better use existing data and guide future testing in toxicology by ITS were elaborated. Based on an earlier OECD workshop (OECD 2008) and earlier work (Jaworska et al. 2010), they delineated the conceptual requirements as being (a) transparent and consistent, (b) rational and (c) hypothesis-driven. Notably, these resonate strongly with the principles of Evidence-based Toxicology (Hoffmann and Hartung 2006).

We have earlier summarized the many reasons for combining tests or test results (Table 1).

**Table 1:** Reasons to combine tests^a^

Not all possible outcomes of interest are covered in a single test
Different modes of action need to be covered, which may cause the same toxicological effect
In vivo processes usually involve a chain of events while one in vitro test often represents only a single or a few steps of this complex process
Not all classes of test substances are covered (applicability domains)
Not all severity classes of effect are covered
The positive test result is rare (low prevalence) and the number of false-positive results becomes excessive
The gold standard test is too costly or uses too many animals and substances need to be prioritized
The accuracy (human predictivity) is not satisfying and predictivity can be improved
Existing data and evidences from various tests shall be integrated
ADME (Absorption, Distribution, Metabolism and Excretion) information shall be integrated to make an in vivo extrapolation from data

^a^Compiled and modified from Hartung et al. (2013) and Rovida et al. (2015)

Stand-alone methods, able to substitute completely for an in vivo test, were the frontier of the past, aiming for a full replacement, and demonstrated through validation (Hartung et al. 2004; Leist et al. 2012) to be largely applicable in specific toxicological test like skin irritation, skin corrosion, or phototoxicity testing, the 3Rs vision must take into account the difficulties to find solutions when it is necessary to mimic multiple physiological responses or complex pathways of toxicity. A possible way for overcoming intrinsic limitation of stand-alone method is the use of a testing battery in which different elements of existing information are assembled through a specific sequence of tests. These test batteries are generally defined as integrated approach in which mechanistic data are combined with different information sources. The integrated approaches most frequently applied in regulatory contest, particularly for complex toxicological endpoints as carcinogenicity or skin sensitization, are the IATA, the DA and the Adverse Outcome Pathways (AOP) (Rovida et al. 2015; Eskes 2019; Leist et al. 2017). Table 2 shows definitions for associated terms. OECD has coined the term IATA and it is, therefore, the term of choice for the field. IATA is based on integration of weighed multiple information and an expert judgment is always requested for regulatory decisions (OECD 2017a), while DA is more standardized being based on a fixed data evaluation (Casati 2018a; Eskes 2019). Conversely, AOPs are only based on mechanistic information; they describe the sequence of events that, starting from an initial perturbation at biological level due to a stressor(s), induce an adverse effect in the organism of regulatory relevance going through a series of intermediate events (Vinken et al. 2017; OECD 2018e). Notably, the usefulness of AOP to design IATA was recognized in an OECD workshop (Tollefsen et al. 2014). A fruitful aspect on evolution of alternative methods and their applicability, is the reconversion of pre-existing test, with the necessary changes, to be adopted in another domain, how has seen recently for the Reconstructed human Epidermis models (RhE), firstly applied in skin corrosion (OECD 2019a), but recently included in phototoxicity (OECD 2021a).

**Table 2:** Definition of reference terms for integrated approaches^a,b,c^

Term	Definition	Comment
Test battery no consensus definition	A series of tests, independent of each other, generally designed to complement each other and/or to measure a different component of a multi-factorial toxic effect, and which are usually performed at the same time or in close sequence. Test batteries typically tend to complement each other but are not integrated into a strategy A group of assays conducted together for a specific purpose, usually to provide a prediction for a toxicity endpoint. The results of each individual assay could be equally weighted, or a statistical weight could be used as an attempt to better model the in vivo response	“which are usually performed at the same time or in close sequence.” not needed Weighing would make it an ITS
Tiered test scheme	Testing approaches based on sequential assessments, where a result at one tier is used to determine the next step, if any. It is usually a decision-tree type of testing; after each step, the information is assessed to determine whether a prediction for the toxicity endpoint can be made or whether further testing/analysis needs to be done. A tiered approach usually progresses from a review of existing literature and data to a review of data for related chemicals or formulations, to perhaps a SAR/(Q)SAR analysis, to simple in vitro screening assays, to the use of more complex in vitro three-dimensional models, to testing in lower species, to the traditional animal test	OECD
Integrated testing starategy [ITS] no consensus definition	A methodology, which integrates information for toxicological evaluation from more than one source, thus facilitating decision-making Strategies to gather and analyze a broad range of data coming from different sources (epidemiological studies, animal data, in vitro data, read-across methodologies, etc.) and used to draw conclusions based on weight-of-evidence approaches Testing strategies composed of, e.g., a number of in vitro and in silico methods that, combined and weighted in a fixed way, would serve to replace some or all in vivo experimentation for a given toxicity endpoint Approaches that integrate different types of data and information into the decision-making process. In addition to the information from individual assays, test batteries, and/or tiered test schemes, integrated testing strategies may incorporate approaches such as weight-of-evidence and exposure/population data into the final risk assessment for a substance	Definition describes evidence integration not necessarily through testing Weight-of-evidence too narrow Focus on replacement too narrow, not necessarily fixed DIP Definition describes evidence integration not necessarily through testing
Defined approach to testing and assessment	A defined approach consists of a fixed data interpretation procedure (DIP) (e.g., statistical, mathematical models) applied to data (e.g., in silico predictions, *in chemico*, in vitro data) generated with a defined set of information sources to derive a prediction. In contrast to the assessment process within IATA, that necessarily involves some degree of expert judgment, predictions generated with defined approaches are rule-based and can either be used on their own if they are deemed to fit-for-purpose or considered together with other sources of information in the context of IATA	OECD
Integrated approach to testing and assessment (IATA)	Approach based on multiple information sources that integrates and weights all relevant existing evidence and guides the targeted generation of new data, where required, to inform regulatory decision-making regarding potential hazard and/or risk. An IATA necessarily includes a degree of expert judgement, for example, in the choice of information sources and their weighting. Nevertheless, some of the IATA components, such as defined approaches to testing and assessment, can be standardised (i.e., rule-based)	OECD
Data interpretation procedure (DIP)	Any fixed algorithm for interpreting data from one or typically several information sources. The output of a DIP is typically a prediction of a biological effect of interest. A DIP is rule-based in the sense that is based for example on a formula or an algorithm (e.g. decision criteria, rule or set of rules) that do not involve expert judgment. This definition has been taken and adapted from OECD guidance document 34	OECD
Information source in the context of IATA	Physicochemical properties (e.g., molecular weight, pK_a_, Log K_ow_ etc.), testing methods (i.e. *in chemico*, in vitro, in vivo methods), non-testing methods (e.g. QSARs predictions, extrapolation from chemical grouping approaches), and any other source that can generate relevant information for the purpose of the assessment within a defined approach or IATA	OECD

*DIP* data interpretation procedure, *IATA* integrated approaches to testing and assessment, *ITS* integrated testing strategy, *Log K*_*OW*_ decadic logarithm of the n-Octanol/water partition coefficient, i.e., a measure of lipophilicity, *OECD* the organisation for economic co-operation and development, *pK*_*a*_ the negative log base ten of acid dissociation constant, i.e., a quantitative measure of the strength of an acid in solution, *(Q)SAR* (quatitative) structure activity relationship, *SAR* structure activity relationship sources

^a^https://www.oecd.org/officialdocuments/publicdisplaydocumentpdf/?cote=env/jm/mono(2016)28&doclanguage=en

^b^http://alttox.org/mapp/emerging-technologies/integrated-testing-strategies-risk-assessment/

^c^Ferrario D. et al. ALTEX 31, 2014

The term IATA has been coined by OECD^1^ (OECD 2008), describing it as *“pragmatic, science-based approaches for chemical hazard characterization that rely on an integrated analysis of existing information coupled with the generation of new information using testing strategies. IATA follow an iterative approach to answer a defined question in a specific regulatory context, taking into account the acceptable level of uncertainty associated with the decision context.”* In the context of the OECD *Guidance Document on the Use of the Adverse Outcome Pathways in developing IATA*^2^ (Series on Testing and Assessment No. 260 2017), an IATA generic structure was defined consisting of three sequential steps/ parts: (i) the collection of existing information relevant for the chemical under evaluation; (ii) the weight-of-evidence evaluation of the gathered information that may enable regulatory decision-making regarding potential hazard and/or risk or may indicate what additional information is needed for a sound assessment; and (iii) the generation of new testing data to support the conclusion. This, in principle, suggests the use of IATA for all hazards. Next, a review of the state-of-the-art of 3Rs approaches in topical and systemic toxicities with specific view on the state of IATA development shall elucidate innovative perspectives for the future of replacement of the traditional test.

## Topical toxicities

### Skin irritation and skin corrosion

Reconstructed human epidermis models (RhE), skin tissue derived from non-transformed keratinocytes cultured on insert polycarbonate support that mimic morphological, physiological and biochemical characteristics of the human epidermis (Gordon et al. 2015), are getting more and more attention, for their wide application, becoming relevant in testing cosmetics, chemicals, and in pharmaco-toxicological research (Faller et al. 2003; Kandarova et al. 2005, 2006, 2018; Netzlaff et al. 2005; Schäfer-Korting et al. 2008; Gordon et al. 2015; Kandarova and Hayden 2021).

In skin irritation and corrosion, defined as a reversible and irreversible skin damage, respectively, RhE, that represent the in vitro target organ, firstly adopted for skin corrosion in 2004 (OECD 2019a), and subsequently in skin irritation in 2010 (OECD 2019b), are applied, with different protocols, as stand-alone methods, and identified as model of total replacement based on the endpoint of cell viability, evaluated with the well-known toxicity test MTT.

In skin irritation, that historically used laboratory animals with the Draize Skin Irritation Test (OECD 2015a), the in vitro RhE Test (OECD 2019b, is the only recognized alternative method, including EpiSkin™ (SM), EpiDerm™ SIT (EPI-200), SkinEthic™ RHE, LabCyte EPI-MODEL24 SIT, epiCS^®^, Skin + ^®^. Actually, these methods represent already very simple testing strategies, because they are preceded by a pH measurement with testing being not necessary if the substance is a strong acid (pH ≤ 2.0) or base (pH ≥ 11.5).

For skin corrosion, two more validated methods, though non-human models, are available: the Rat Skin Trans Cutaneous Electrical Resistance (TER) (OECD 2013), an ex vivo*/*in vitro test, based on the use of skin from rats at 28–30 days humanely killed, where the endpoint is the alteration of the skin barrier, and the Corrositex^®^ assay, (OECD 2015b), that is based on the use of an artificial biomembrane, and the endpoint is the barrier damage, caused by corrosive substances, acidic and alkaline.

An implementation of RhE through new technologies like automation or bioprinting, the improvement of the model through for example the partial or total elimination of animal components from the medium, to allow the application of microfluidic techniques, could bring to a broader application of the RhE model in regulatory pharmacology and toxicology, but also in personalised medicine (Kandarova et al. 2021). Moreover, the RhE models, found application in other domains, as demonstrated recently for phototoxicity (OECD 2021a), genotoxicity (Pfuhler et al. 2021), skin sensitization (McKim et al. 2012; Saito et al. 2013; Johansson et al. 2019) and various pharmacological applications.

The methods for skin irritation and corrosion testing have been combined to an IATA in the context of the European REACH legislation and the OECD (2014a). In vivo testing is foreseen only as a last resort for example in those cases where in vitro methods are not suitable for testing the specific substance or if the results of the in vitro tests are not adequate for the regulatory need.

### Phototoxicity

Photoxicity is classified as an acute toxic effect due to the activation of photoreactive chemicals by cutaneous exposure to UV or visible light. Exposure to photoreactive chemicals can occur both by topical application (UV-filters, cosmetics) and by systemic administration (drugs). These light-mediated effects may be in turn categorized as photoirritation (acute light-induced skin response to a photoreactive chemical), photoallergy (immune-mediated reaction), and photogenotoxicity (genotoxic response either directly by photoexcitation of DNA or indirectly by excitation of photoreactive chemicals). Assessment of photoxicity (mainly as photoirritation) is requested for cosmetics and drugs. Three OECD Testing Guidelines (TG) based on in vitro assays are currently available: TG 432, based on Neutral Red Uptake (NRU) by 3T3 mouse fibroblast (OECD 2019c), TG 495 based on the evaluation of Reactive Oxygen Species (ROS) formed by chemicals irradiated with a simulated sunlight (OECD 2021b), and TG 498 based on the use of reconstructed human epidermis tissues (OECD 2021a). The in vitro 3T3 NRU Phototoxicity Test was the first in vitro test included in an OECD guideline; it compares cytotoxic effect of a test substance, determined by the relative reduction in cell viability exposed to test chemical, in presence or in absence of light. Several limits have been ascribed to this test as the lack of bioavailability/biokinetics modelling resulting in poor correlation in vivo-in vitro (Onoue et al. 2013) and the high frequency of false-positive results (85% of in vitro 3T3-NRU positive assays were negative in further in vivo testing) (Lynch and Wilcox 2011) but it is generally considered robust enough. Moreover, the use of a mouse cell line has been criticized and the substitution of 3T3 cell line with human keratinocytes was proposed, to provide a more realistic experimental model with respect to the human situation (Clothier et al. 1999; Maciel et al. 2019). To date, 3D human skin models represent a flexible tool to investigate skin alterations after chemical exposure, including phototoxicity, since they take into account also some relevant in vivo parameters as skin penetration or stratum corneum barrier function (uco Dayane et al. 2018; Kim et al. 2020). In this respect, the new OECD TG 498 (OECD 2021a) fully reflects these aspects; it is based on the in vitro test system of the RhE, which closely mimics biochemical and physiological properties of the human epidermis (Dellambra et al. 2019). Moreover, the test system uses human-derived keratinocytes to reconstruct an epidermal model which maintains histology and cytoarchitecture of the human skin. This test guideline is applicable to determine the phototoxic potential of test chemicals after topical application to RhE tissues in presence and absence of simulated sunlight. Phototoxicity potential is evaluated by viability reduction of RhE tissues exposed to the test chemicals. No IATA developments were identified for the phototoxicity hazard. Potentially, the fibroblast and the RhE models could be combined into an IATA. Furthermore, the combination with genotoxicity assays for photogenotoxicity or with skin sensitization assays would be possible, which is, however, these are no standard testing requirement.

### Skin sensitization

A skin sensitizer is defined a chemical substance or mixture that can induce an allergic response at the skin level (i.e., allergic contact dermatitis, ACD) after repeated dermal exposure. Currently, more than 3000 chemicals were classified as skin sensitizer (de Ávila et al. 2019). Often skin allergens have electrophilic moieties which ensure their reaction with the nucleophilic sites in skin proteins. In the current regulatory framework, skin sensitization is mandatory for many consumer products and related ingredients as cosmetics or agrochemical products. In recent years, OECD promoted some in vitro approaches addressing different mechanistic events of the skin sensitization process summarised in an AOP (OECD 2014a, b; Basketter 2016). In this AOP four sequential key events (KE) have been identified:

(a) KE-1 (defined also as Molecular Initiating Event, MIE) represents the covalent irreversible binding of electrophilic substances to nucleophilic sites of dermal proteins producing an antigenic stimulation of human immune system. KE-1 is covered by the Direct Peptide Reactivity Assay–DPRA, (OECD 2021c) in which the reactivity of test chemicals towards model synthetic peptides containing either lysine or cysteine are quantified.

(b) KE-2 represents inflammation of keratinocytes, the most common epidermal cells. Methods corresponding to this step are the KeratinoSens™ and the LuSens tests, both based on a specific cell signalling pathway of the antioxidant/electrophile response element (ARE)-dependent pathway, comprised in the ARE-Nrf2 Luciferase Test Method, (OECD 2018a). Luciferase-based reporter assays are highly sensitive and easy to use. Moreover, interferences are rare and usually restricted to direct structural interactions of test substance with the luciferase enzyme.

(c) KE-3 represents the activation of dendritic cells (DC) detected by expression of specific cell surface markers, as chemokines and cytokines, whose maturation and migration to lymph nodes provides the essential trigger for the following KE-4. Assessment of the KE-3 is quite challenging due to the complex biology of DC activation. Three assays addressed this endpoint: (i) the human cell Line Activation Test or h-CLAT method, (ii) the U937 Cell Line Activation Test or U-SENS and (iii) the Interleukin-8 Reporter Gene Assay or IL-8 Luc assay, (OECD 2018b). All these test methods both quantify changes in the expression of cell surface marker(s) associated with the DC activation after exposure to sensitisers (as CD54, CD86) and changes in cytokine IL-8 expression, also associated with DC the activation.

(d) KE-4 represents T cell proliferation and activation. This is the most complex event closely related to immune system response in vivo; so far, no validated in vitro methods are available for this endpoint, so it is generally addressed by the murine Local Lymph Node Assay, LLNA, (OECD 2010a), a validated refinement and reduction alternative in mice.

It is important to highlight that the in vitro tests of KE 1–3 if used alone are not considered to provide a level of information for risk assessment comparable to in vivo tests (e.g., LLNA assay) since they are considered insufficient to cover the complexity of the biological mechanisms occurring in vivo. Therefore, results obtained with these methods have to be used in conjunction with other relevant information, as physico-chemical properties, in IATA or DA framework (Casati 2018a; b; Kleinstreuer et al. 2018). Twelve case studies of Das were submitted to OECD including DA with fixed data interpretation procedures as well as IATA incorporating expert judgment.

Skin sensitization therefore pioneered IATA development: A generic IATA for skin sensitisation was proposed in the European Chemicals Agency’s (ECHA) Guidance to Industry on Information Requirements and Chemical Safety Assessment (Chapter R 7.a, section R.7.3 Skin sensitisation). The guidance was revised to comply with the changes made to the REACH legal text adopted in Version 5.0 2016 making the use of in vitro methods for skin sensitisation testing a standard information requirement and the primary choice over in vivo studies. Several integrated approaches have been constructed for skin sensitisation depending on the final regulatory purpose. With the aim to harmonize the different approaches and to produce at least the same level of information of LLNA assay for hazard identification (i.e., discrimination of sensitising substances from non-sensitizers) a Guideline Document on Defined Approaches for skin sensitization has been recently published as OECD TG 497 (OECD 2021d). Three DAs are included in this Guideline, two of them are also able to provide information for sensitisation potency categorisation, equivalent to the potency categorisation provided by LLNA. In this process, Kleinstreuer et al. (2018) developed generic evaluation categories and criteria for DA (Table 3).

**Table 3:** Qualitative evaluation categories and criteria for Defined Approaches^a^

Evaluation category	Evaluation criteria
Characteristics	Principle Prediction (i.e., hazard versus potency [categories or continuous]) Publication Information sources
Input data	Test method (in vitro and *in chemico*) Read-out used Validation status Reproducibility Issues (e.g., IP, availability) In silico/expert system data/physicochemical properties Read-out used Availability Reliability Issues (e.g., IP, availability) Expert knowledge Input used Availability Principle Prediction (i.e., hazard vs. potency [categories or continuous]) Publication Information sources
Prediction algorithm	Type Availability Transparency Requirements for implementation (specific software) Self-learning Complexity Sequential information generation All inputs required? Predictivity: Sample size (total and for categories) Predictivity: Parameters (sensitivity, specificity, concordance)
Mechanistic relevance	OECD AOP key events covered Sequence of OECD AOP events considered Justification/discussion of the mechanistic relevance
Applicability domain	Chemical spectrum tested Limitations (solubility, surfactants) Potential limitations for cosmetic ingredients (e.g., natural extracts cannot be processed by in silico approaches)
Practical aspects	Costs Can be conducted by CRO [contract research organization]? Time required (per substance)

^a^https://echa.europa.eu/documents/10162/13632/information_requirements_r7a_en.pdf/e4a2a18f-a2bd-4a04-ac6d-0ea425b2567f

The evaluation criteria in Table 3 from Kleinstreuer et al. (2018) are only brief bullets, which need further details. For example, costs can refer to costs per test, cost of infrastructure, labour, or time. The original publication gives more details, but the development of Defined Approaches and its evaluation is still its infancy and represent a challenge to the validation bodies.

Moving forward to skin sensitization process, the AOP developed for this endpoint also contribute to improve the understanding of mechanisms related to other immune-mediated processes such as chemical respiratory allergy, which share some toxicity pathways with skin sensitization, such as T lymphocyte activation and proliferation (Kimber et al. 2018).

### Skin absorption

Skin penetration is considered relevant for occupational and public health risk assessment of specific classes of industrial chemicals such as pesticides and biocides. An OECD TG for an in vitro test for skin absorption was adopted in 2004 (OECD 2010b) and, more recently, OECD released Guidance Notes to facilitate the harmonization of the experimental data from absorption studies (OECD 2019d). TG 428 (OECD 2010b) is based on absorption of a test substance applied to the surface of a skin samples separating the two chambers (donor chamber and receptor chamber) of a glass diffusion cell. Both static and flow-through diffusion chamber are acceptable. Skin samples, to a specific thickness, from human or animal sources can be used. Normally, viable skin is preferred but standardized non-viable skin can also be used checking its integrity prior use. Since human skin is considered less permeable than that of laboratory animals (i.e., rodents), data obtained with human skin samples are considered as stand-alone data to predict the expected absorption in humans. Combination of animal, human in vitro*,* and human in vivo data is also suggested; for example, the “Triple Pack approach” combines three types of dermal absorption data derived from: (1) in vivo animal; (2) in vitro animal; and (3) in vitro human dermal absorption studies (EFSA Panel on Plant Protection Products and their Residues [EFSA PPR] 2011).

Suitability of commercial reconstructed human epidermis model and/or full human skin equivalents (i.e., including both epidermal and dermal layers) for absorption studies has been investigated with chemicals and drugs. Results show that these models are more permeable than human skins (Henning et al. 2009). These limited barrier properties are mainly attributable to differences in composition and non-homogeneous distribution of lipids that hampered the formation of a continuous lipid barrier (Tfayli et al. 2014). Moreover, absence of the vascular network also plays a critical role in the establishment of barrier functions. For this reason, the use of these skin models for absorption studies is still limited (Neupane et al. 2020).

Simple and reproducible alternatives to human and animal skins are also represented by synthetic artificial membranes (e.g., multi-layered silicone-based membranes) (Abd et al. 2016). They may be easily procured and stored and show less variability than biological skins. Conversely, because of the lack of the superficial barrier of the stratum corneum, they show a poor correlation with human absorption data. So, they are recommended for the initial screening of new molecules while permeation data for hazard assessment should be obtained on biological skin models (Neupane et al. 2020). No IATA developments were identified for this aspect of toxicokinetics (Tsaioun et al. 2016), notably not a hazard. In general, biokinetic (ADME) represent an enormous opportunity for IATA development.

### Eye irritation/corrosion

For the evaluation of eye irritation and serious eye damage, historically one of the priorities of alternative methods (Adriaens et al. 2014), no stand-alone test is available, but combined methods in a testing strategy, with a top down and a bottom-up approach, were described (Scott et al. 2009), to replace the in vivo ocular Draize rabbit test (OECD 2017b), where the damage in iris, conjunctiva and cornea is scored.

To date, several NAMs are validated for testing singular chemicals but with limited evidence for mixtures of compounds. Non-human tests, with different endpoints, have been validated like the ex vivo*/ *in vitro methods of the Bovine Corneal Opacity and Permeability test (BCOP) (OECD 2017c) and the Isolated Chicken Eye test (ICE) (OECD 2018c), where the application of the 3Rs is firstly evident in the ethical sourcing of the collected eyes from slaughterhouses, and further in vitro tests like the Fluorescein Leakage (FL) Test method, (OECD 2017d), a cell based assay with the Madin-Darby Canine Kidney (MDCK) cells and fluoresceine as marker, the Short Time Exposure (STE) In Vitro Test Method (OECD 2020a), with Staten Seruminstitut Rabbit Cornea cells (SIRC), and the in vitro macromolecular test method, (OECD 2019e). The microphysiometer method was validated for non-irritant substances (Hartung et al. 2010) but so far not taken up in an OECD test guideline.

The Reconstructed human Cornea-like Epithelium (RhCE) model, a human-based 3D model (OECD 2019f) mimics the human corneal epithelium. It is an in vitro test for chemicals not requiring classification and labelling for eye irritation and serious damage, like Vitrigel^®^-EIT (OECD 2019g) another model based on immortalized cornea cells.

Different commercial tissues of RhCE are reported in the OECD 492 (OECD 2019f), like EpiOcular™ EIT, SkinEthic™ Human Corneal Epithelim HCE EIT, LabCyte CORNEA-MODEL24 EIT, and MCTT HCE™ EIT, are now available (Kolle and Landsiedel 2021).

Even if these models have many advantages and domains of application, there are also some limits that draw attention: one emerging problem, to solve in the near future regulatory requirements, is testing of agrochemical formulations, that even if they fall in the definition of “mixture”, included in OECD guidelines, there are no available NAMs until now for the evaluation of serious eye damage or for skin irritation potential (Kolle and Landsiedel 2021).

IATA development for eye irritation and corrosion has progressed very much in parallel to skin irritation and corrosion with the difference that there is less agreement whether and how the different NAMs in combination can avoid animal testing. The different OECD methods are broadly accepted to identify either non-irritant or corrosive but for irritant substances there is only a weight-of-evidence approach. An IATA has nevertheless been put forward by OECD (2017e). Casati (2018a) concluded “*Classification of GHS Category 2 chemicals (eye irritation) can be concluded only with a weight-of-evidence approach. Thus, the decision process within the IATA for serious eye damage and eye irritation IATA cannot be fully standardised at the moment and for this reason, there is no assurance for international use and acceptance of *in vitro* data in cases where conclusions are derived on the basis of weight-of-evidence*”.

## Systemic toxicity

### Genotoxicity

Genotoxicity refers to the ability of chemical agents to damage genetic information within the cells causing mutations and/or inducing alterations in the DNA molecules, as modifications in nucleotide sequence or in double helix structure. While all mutagenic substances are genotoxic, not all genotoxic substances are mutagenic. Hence, to perform genotoxicity hazard evaluation of chemicals three different endpoints are requested at least, one for mutagenicity, as in vitro gene mutations assays, and two for DNA alteration, as chromosomal aberrations (clastogenicity), and chromosomal damage which can cause a change in their number (aneuploidy). Hence, a test battery is requested utilizing in vitro approaches followed, in some cases, by in vivo confirmation when positive results are reported in vitro. In vitro assays for genotoxicity have been validate for long time and are suitable to identify direct chemical effects on DNA while secondary genotoxicity effects (i.e., mediated by the immune system) are not resolved by standard in vitro approaches so far. A standard approach for in vitro genotoxicity includes: (i) a gene mutation test in bacteria (OECD 2020b) or in mammalian cells using two different gens (OECD 2016a, b) and (ii) a structural chromosomal test, as the in vitro mammalian chromosomal aberration test (OECD 2016c) and the in vitro micronucleus test (OECD 2016d) that identify micronuclei in the cytoplasm of interphase cells. The latter is a very popular and applied test, suitable to detect both clastogenic and aneugenic effects. Moreover, albeit not regulated by specific TGs, detection of DNA damage (as single and double strand breaks or specific DNA lesions) can provide useful information about chemical genotoxic potential. In this respect, the in vitro Comet assay (with or without incorporation of Formamidopyrimidine DNA glycosylase (Fpg)) is widely used to determine DNA effects due to oxidative stress (Kohl 2020). It has been validated (though not peer-reviewed by a validation body yet) in vitro using the human lymphoblastoid TK-6 cell line (Muruzabal et al. 2021) for 3D reconstructed human skin (Pfuhler et al. 2021). One of the main shortcomings of in vitro genotoxicity assays is that they frequently produce false-positive results, due to the coupling of high sensitivity with a relatively low specificity (Corvi and Madia 2017). The combination as a test battery where any positive test results in a positive call accumulates these.

OECD guidelines for in vitro genotoxicity testing are mainly focused on 2D models but, recently, increasing efforts have been channelled to the use and optimization of in vitro 3D models. In particular, robust protocols for genotoxicity assessment have been established for both Micronucleus and Comet assay on 3D models representative of different routes of exposure, i.e., dermal (commercial skin models), inhalation (airway model) and systemic (mainly liver spheroids) (Kooter et al. 2016; Wills et al. 2016; Shah et al. 2018; Conway et al. 2020). However, some experts believe that these assays are not mature enough to develop specific TGs (Pfuhler 2020). The improving of efficiency of in vitro genotoxicity assays is another point that need to be stressed. Development of high-throughput methodology as well as assay miniaturization (96-well microplate-based and chip-based) are considered a priority to produce a large quantity of genotoxicity data in a fast and cost-effective way (Guo et al. 2020).

With current proposals to revise the European REACH prompting in vivo confirmation of positive in vitro mutagenicity findings,^3^ IATA development to better interpret in vitro results and possibly include steps before moving to in vivo is most promising.

### Carcinogenicity

Carcinogenesis is a multistep process in which transition of normal cells into cancer cells is mediated by a sequence of biological events. Several major hallmarks of this process have been identified including genome instability, inflammation, immunosuppression, and metabolism deregulation (Madia et al. 2021). Evaluation of genotoxicity is also the preliminary step for determination of carcinogenic potential of chemicals. This is a multi-step process that involves complex biological interactions driven by many different factors (genetic, hormonal, age related, environmental, etc.), so it is extremely difficult to address this endpoint by stand-alone in vitro approaches. The in vitro cell transformation assay (CTA) covers some of the key phases of the carcinogenicity process; it was not considered robust enough for an OECD TG but it was reported in two OECD Guidelines, Syrian Hamster Cells (SHE) CTA (OECD 2015c) and Bhas 42 cell line CTA, (OECD 2016e). To date, the use of IATA is strongly recommended for carcinogenicity evaluation (Eskes 2019; Dal Negro et al. 2018) particularly for non-genotoxic carcinogens where a dedicated expert group was established at OECD in 2014 (Corvi et al. 2019; Jacobs et al. 2020). OECD recognising that the CTA alone was insufficient to address non-genotoxic carcinogenicity and that a more comprehensive battery of tests addressing different non-genotoxic mechanisms of carcinogenicity would be needed in the future, identified the need for an IATA to properly address the issue of non-genotoxic carcinogenicity. The expert working group examines the current international regulatory requirements and their limitations in respect to non-genotoxic carcinogenicity, and how an IATA could be developed to assist regulators in their assessment of non-genotoxic carcinogenicity. The development of an ITS is ongoing also in the context of the International Conference on Harmonisation (ICH), the collaboration of pharmaceutical regulators, based on knowledge of pharmacological targets and pathways, together with toxicological and other data. The European Partnership for Alternative Approaches to Animal Testing has started a project to evaluate whether this approach is applicable to the carcinogenicity assessment of pesticides. The promise of IATA for carcinogenicity was recognized in our roadmap exercise (Basketter et al. 2012; Leist et al. 2014). It is exciting to see that several high-level projects are now realizing this vision.

IATAs for carcinogenicity will require the combination of various tests as in vitro genotoxicity and carcinogenicity assays are able to address just some stages of the respective in vivo process that are characterized by multi-step components. To date, the in vitro tests cover specific and basic cellular endpoints (such as gene mutation, chromosomal damage, cell transformation) and no cell signalling or gene expression are considered, though these are increasingly considered as valuable biomarkers.

### Developmental and reproductive toxicology

A very high number of laboratory animals is involved in studies for reproductive toxicology (Hartung and Rovida 2009) with many false-positives (Hartung 2009) as well as low inter-species concordance (Smirnova et al. 2018) and in the last years many alternative methods to the in vivo studies were set up and evaluated (Brannen et al. 2016; Corvi et al. 2019).

Three alternative methods, even if not accepted for full replacement, are available (Adler et al. 2011; Pistollato et al. 2021): the mouse Embryonic Stem Cell test (EST), a cell-based assay, Micromass Test (MM), the Whole Embryo Culture (WEC), based on embryonic tissue, limb and cephalic tissue, and the Whole Embryo Culture (WEC), on rat embryos (Pistollato et al. 2021).

The toxicological effects of xenobiotics on EST, is based on three different endpoints: the inhibition of differentiation on Embryonic Stem Cells (D3) and proliferation, through a cytotoxicity test, either on ESC (D3) and 3T3 fibroblasts adult cells, (Spielman et al. 2006; Brannen et al. 2016).

Even if EST is considered a model with a low biological complexity (Brannen et al. 2016), there are many advantages since the cells could be maintained in vitro, reducing costs and it can be applied in high-throughput screening (Brannen et al. 2016), or as a part of an integrated strategy (Pistollato et al. 2021).

We were not able to identify any attempts to establish IATA for reproductive toxicology in the regulatory arena, while such an approach was highly recommended in our roadmap exercise (Basketter et al. 2012; Leist et al. 2014). The EU projects ReProTect (Hareng et al. 2005) and ChemScreen (van den Burg et al. 2011) developed such a preliminary IATA, with promising results (Schenk et al. 2010). Given the resource-demanding impact of this hazard, it is not clear why so little effort is put into developing an IATA for developmental and reproductive toxicology.

### Endocrine disruptors

The different and multiple mechanisms of action and pathways of Endocrine Disruptors, and MIEs, imply a complex approach for their toxicological evaluation (OECD 2018d), and, to decrease the number of animals used, the Level 2 of testing strategy, based on in vitro approach, should be implemented with regulatory validated methods. In vitro tests currently available, are focused on the action of chemicals on estrogen receptor agonists and antagonists (OECD 2021e), estrogen receptor binding (OECD 2015d), androgenic receptor agonists and antagonists (OECD 2020c), and on steroidogenesis (OECD 2011).

Future directions and perspectives are oriented to cover other toxicological endpoints, enlarging the field of action, with, for example, assays based on other hormone receptors, or specific target organ effects (Pistollato et al. 2021), and applying methodologies for a large-scale testing, like high throughput screening.

Based on US EPA ToxCast data, which tested about 2000 chemicals in hundreds of robotized assays, pioneering work showed how to use a combination of tests to predict endocrine activity.

(Browne et al. 2015; Kleinstreuer et al. 2018; Judson et al. 2020), which was accepted for the US Endocrine Disruptor Screening Program (EDSP). The respective prediction model is clearly an IATA. So far this has only been applied to estrogenic and androgenic endocrine disruption but it shows the potential of combining a number of tests.

The status of IATA development for different human hazards is summarized in Table 4

**Table 4:** Status of IATA development for different hazards

Hazard	Status	Comment
Skin irritation and skin corrosion	Defined for REACH and by OECD	Only generic IATA
Phototoxicity	None identified	Potential combination fibroblasts with skin models
Skin sensitization	IATA with defined approaches accepted by OECD	The “poster child” of DA/IATA development; challenge of quantitative IATA
Skin absorption*	Non identified	IATA for biokinetics represent an opportunity
Eye irritation/corrosion	Defined for REACH and by OECD	GHS Category 2 (eye irritation) not satisfactorily identified
Genotoxicity	Test batteries in common use, no IATA; OECD guidance to include in other short-term animal studies	Tremendous potential for IATA development (reduce false-positives; step between in vitro and animal test)
Carcinogenecity	Under development OECD, ICH and EPAA project; combination of in vivo cancer bioassay with chronic animal test (TG 453)	Enormous potential because of recognized shortcomings of the animal test
Developmental and reproductive toxicity	None identified beyond EU projects; some combinations of repeated-dose and developmental toxicity animal studies (TG 422)	Enormous potential because of the costs and high animal use
Endocrine disruption	OECD tiered testing strategies but no IATA	Major need for the different endocrine disrupter screening programs and REACH revision

*ICH* international conference on harmonisation, *EPAA* European partnership for alternative approaches to animal testing

*No hazard on its own

## In vivo testing as part of IATA

With the promise of REACH to further also the use and availability of alternative methods, animal tests were usually only placed as last resort in the IATAs developed. However, we should note first of all that the mere combination of animal tests into one can save animals used (Hartung 2018). For example, short-term toxicity tests with rodents can be combined with developmental toxicity screening assays as OECD Test Guideline 422. Similarly, chronic toxicity studies can be combined with carcinogenicity studies in rodents as OECD Test Guideline 453. A number of genotoxicity tests can be incorporated into acute and short-term in vivo assays. The potential of addressing endocrine disruptor effects within guideline studies needs to be further explored. The problem of multiple testing adding more endpoints to these studies is, however, an important caveat (Hartung 2013). The entire area of lower species such as zebrafish, *C. elegans* or Drosophila represent further in vivo assays as building blocks for IATA. Last but not least, legacy data of the past represent valuable information not only for the substances themselves but through read-across (Ball et al. 2016) also for similar chemicals.

## Progress made in recent years and challenges for future developments toward IATA

IATA are slowly but continuously being embraced in regulatory toxicology as shown for the different hazards above. Major driving forces are the European REACH legislation as well as the US EPA’s ToxCast and EDSP program. The fact that OECD has taken over developing guidance for IATA and DA is of critical importance. OECD has for example developed *General Principles for the Reporting of Defined Approaches to Testing and Assessment based on Multiple Information Sources* to facilitate their regulatory use. They request a DA to be based on a fixed DIP and a defined set of information sources, associated with the following set of information:

1. A defined endpoint
2. A defined purpose
3. A description of the underlying rationale
4. A description of the individual information sources used
5. A description of how data from the individual information sources are processed
6. A consideration of the known uncertainties

This reminds very much of the requirements for test definition for the validation of alternative methods (Hartung et al. 2004).

**Fig. 1:**
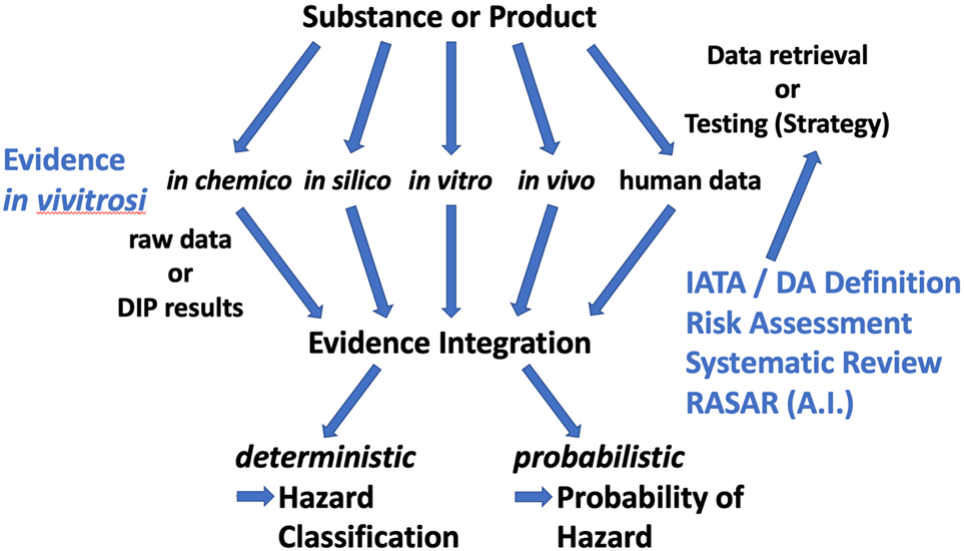
Evidence Integration for Hazard Assessment within IATA, Risk Assessment, Systematic Reviews and Read-Across-based Structure/Activity Relationships Schematic illustrating the similarity of combining various evidence streams for deterministic or probabilistic hazard assessment. Abbreviations (see Table 1 for OECD definition): *DIP* data interpretation procedure, i.e., fixed algorithm for interpreting data to derive test result, *IATA* integrated approach to testing and assessment, *DA* defined approach, *RASAR* read-across-based structure/activity relationship, *A.I* artificial intelligence, aka Machine Learning

An important development was the realization that the fundamental problem of evidence integration from different evidence stream is shared in IATAs, risk assessments and systematic reviews (EFSA 2018), the first prospectively and the latter two retrospectively (Fig. 1). The scheme applies to individual substances such as chemicals and final products (mixtures). Sure, not all evidence generating methods are applicable for the latter, but to some extent this hold also for individual substances (applicability domains). The distinction between prospectively and retrospectively is, however, blurring as more and more existing data are considered in testing strategies and risk assessments/systematic reviews also map testing needs. The advent of machine learning (artificial intelligence, AI) has provided us also with a new tool to do exactly this. This approach is uniquely suited to train on heterogenous datasets, which is called transfer learning. AI makes sense of Big Data (Hartung 2016), which are beside the volume and velocity they are acquired, defined by their variety (forming the 3 V of Big Data). Our earlier work showed, how 74 properties of chemicals could be used to predict hazard classification actually outperforming animal tests (Luechtefeld et al. 2018a, b). For IATA, this has not been sufficiently employed, also because we usually do not have enough training data from NAMs. This has changed with ToxCast and Tox21 and it is most promising to use such information as a source for probabilistic hazard and risk assessment (Maertens et al. 2022).

Schematic illustrating the similarity of combining various evidence streams for deterministic or probabilistic hazard assessment for individual substances or final products (mixtures). Abbreviations (see Table 1 for OECD definition): DIP = Data Interpretation Procedure, i.e., fixed algorithm for interpreting data to derive test result, IATA = Integrated Approach to Testing and Assessment, DA = Defined Approach, RASAR = Read-Across-based Structure/Activity Relationship, A.I. = Artificial Intelligence, aka Machine Learning.

Kinsner-Ovaskainen et al. (2009) gave a number of challenges for ITS/IATA:

- Scientific knowledge and guidance on how to develop an ITS; how to combine the different building blocks for an efficient and effective decision-making process?
- The extent of flexibility in combining the ITS components;
- The optimal combination of ITS components (including the minimal number of components and/or combinations that have a desired predictive capacity);
- The applicability domain of single components and the whole ITS;
- The efficiency of the ITS (cost, time, technical difficulties)
- Need to further discuss and to develop the ITS validation principles

These aspects were discussed in Hartung et al. (2013) but not much conceptual progress has been noted since. With respect to validation, the development of evaluation criteria (Table 3) coupled with performance assessment for the overall DA (Kleinstreuer et al. 2018) was mentioned.

Jaworska and Hoffmann (2010) noted *“Because high-throughput datasets may suffer from technical and biological noise or from various technical biases and biological shortcomings, improved statistics are needed for the separation of signal from noise, as well as for better data integration annotating biologically relevant relationships. The logical interpretation of the complex signal propagation leading to an observed effect is not easily comprehensible. Therefore, computational modelling can be expected to play a crucial role in predicting the output from the signal input or system perturbation to obtain a more comprehensive, less technically biased and more accurate view of the true effect”.* This call for considering the key role of data management (Wilkinson et al. 2016).

The ITS workshop (Rovida et al. 2015) noted that ITS development for hazard characterization in the context of REACH are prescribed, deterministic and serve classification, while ITS for full safety assessment should be flexible, probabilistic, and fit for purpose. We have recently addressed the important role of probabilistic approaches in general (Maertens et al. 2022). The challenges and opportunities for IATA thus have not changed very much since 2015. Some progress for individual hazards is noted as summarized in Table 3; the tremendous opportunity of this approach, however, have not yet been leveraged.

For the improvement of safety sciences and the replacement of traditional animal-based approaches by IATA, approaches should be relevant combining the different aspects of complexity, from chemicals to models. From a multi-disciplinarity perspective, already existing models coming from different scientific domains could be reconverted and applied in toxicological studies and, at the same time, toxicology could be a source for novel methodologies for other fields for cross-cutting applications to speed up the 3Rs vision.

## Abbreviations

AI: Artificial intelligence
AOP: Adverse outcome pathways
ADME: Absorption, distribution, metabolism and excretion
DA: Defined approaches for testing and assessment
DIP: Data interpretation procedure
KE: Key events
IATA: Integrated approaches for testing and assessment
ITS: Integrated testing strategies
LLNA: Local lymph node assay
MIE: Molecular initiating event
NAM: New approach methods
QSAR: Quantitative structure–activity relationships
RhE: Reconstructed human epidermis models
SAR: Structure–activity relationships
TG: Testing guidelines

## Footnotes

1 https://www.oecd.org/chemicalsafety/risk-assessment/iata-integrated-approaches-to-testing-and-assessment.htm.

2 https://www.oecd.org/chemicalsafety/guidance-document-for-the-use-of-adverse-outcome-pathways-in-developing-integrated-approaches-to-testing-and-assessment-iata-44bb06c1-en.htm.

3 https://echa.europa.eu/-/clarification-to-degradation-and-mutagenicity-testing-under-reach.
